# Evaluation of bacteriostatic potency of expired oral paediatric antibiotics and implications on infant health

**DOI:** 10.11604/pamj.2014.19.378.2156

**Published:** 2014-12-15

**Authors:** Adenike Ogunshe, Patience Adinmonyema

**Affiliations:** 1 Applied Microbiology and Infectious Diseases, Department of Microbiology, Faculty of Science, University of Ibadan, Ibadan, Nigeria

**Keywords:** Antibiotic resistance, drug allergy, drug degradation, drug toxicity, expired antibiotics, infant mortality, paediatric antibiotics, paediatric health

## Abstract

**Introduction:**

In spite of significant risks, as well as non-clinical importance due to loss of potency, stiff penalties against administration of expired medications are still not appropriately enforced by health policy makers in many developing countries, possibly because of little evidence to support that expired medications are hazardous. The purpose of this study therefore, was to investigate the effect of expiration dates on in vitro bacteriostatic potentials of oral paediatric antibiotics.

**Methods:**

Comparative bacteriostatic potentials of 31 expired and seven corresponding unexpired oral paediatric antibiotics were determined on infantile diarrhoeagenic bacteria, using a modification of agar well-diffusion method.

**Results:**

Verall total percentage in vitro resistance rates against expired and unexpired paediatric antibiotics respectively were - E. coli (≤100% vs. ≤15.9%), Klebsiella pneumoniae (≤100% vs. ≤31.3%), Proteus mirabilis (≤91.7% vs. ≤41.7%) and Staphylococcus aureus (≤100% vs. ≤18.2%). Resistance rates of 45.5-55.8% (sulfamethoxazole + trimethoprim 5), 39.5-63.6% (amoxycillin 6), 46.5-54.5% (cotrimoxazole 7), 37.5-63.6% (ampicillin + cloxacillin 18), and higher resistance rates of ≥75.0-100% were exhibited towards remaining expired antibiotics. Higher total resistance and multiple antibiotic resistance (MAR) rates were also recorded against expired antibiotics (45.2-93.5%) compared to unexpired antibiotics (28.6-57.2%), except for few strains of E. coli and Proteus mirabilis. Furthermore, unexpired paediatric antibiotics exhibited wider zones of inhibition towards the test diarrhoeagenic bacteria (≥25.0 mm diameter).

**Conclusion:**

This study provided preliminary microbiological results on the appreciable reduction in in vitro bacteriostatic potentials, as well as higher resistance and multiple antibiotic resistance rates among expired oral paediatric antibiotics on infantile diarrhoeagenic bacteria. Apart from less-efficacy, administration of expired antibiotics can lead to increased antibiotic resistance and clinical treatment failure, as well as adverse drug reactions.

## Introduction

Little is known about the general trends of antibiotic resistance in infantile diarrhoeal cases in developing countries like Nigeria, where diarrhoea is a major public health problem. However, it is commonly believed that the relationship between antibiotic use and emergence of antibiotic resistance, as well as the spread of antibiotic resistance is complex. Similarly, there is growing universal concern regarding counterfeit medications, and in particular, counterfeit antimicrobial drugs, which are a threat to public health, with many devastating consequences for patients, including increased mortality and morbidity [1]. Poor qualities of antibiotics and lack of quality compliance and monitoring have also been reported to account for antibiotic resistance in clinical infectious cases [2–8], while counterfeit / adulterated antibiotics or those in which the active ingredients have been replaced by less expensive alternatives also constitute adversely to antibiotic resistance [1, 8–14].

Expired antibiotics in spite of their numerous hazards [15] are however, mostly not seriously considered as having serious adverse health effects on humans, especially with regards to clinical treatment failures. Drug expiration date, which is mostly two to three years after the drug is manufactured, except in some cases, is the specific date until which the medication will be effective, if stored under proper conditions of light, temperature and moisture but if not stored under suggested conditions, the medications may lose potency even before the expiration dates. Whereas, in spite of warnings that medications that have passed their expiration can pose significant risk, and that it is extremely important to properly dispose of expired medications to avoid serious injuries to older adults and children [16], not everyone still agrees that expired medications are unsafe. It has even been reported that many drugs stored under reasonable conditions retain 90% of their potency for at least five years, and sometimes much longer, after the expiration date on the label [16], while some further claimed that expiration date is a very conservative marketing ploy by drug manufacturers to keep consumers in restocking their medicine cabinets [17].

Expiry dates provided by manufacturers are however, essentially a guarantee that the stated level of active ingredients will be present at least until the expiry date [18]. Whereas, some pharmacologically active drugs produced in industrialised countries have expired prior to distribution in developing countries, and sometimes, expired drugs may receive new labels and be dumped into developing countries without label or be donated rather than sold [19–21]. In some developing countries, the authenticity of information on the packages of some imported drugs and drug products cannot be fully ascertained [22]. In addition, a common menace by unscrupulous drug merchants in Nigeria is that expiration dates on the medications are sometimes replaced with fake dates that usually further extended the already expired dates by additional years; thus, expired clinical drugs are deceitfully sold and administered. Meanwhile, one of the dangers of taking expired antibiotics is that over time, they not only lose their chemical integrity and efficacy but could play a significant role in creating antibiotic resistance. The aim of this preliminary study therefore, was to investigate the in vitro bacteriostatic potency of expired, oral paediatric antibiotic suspensions that are commonly available in Nigeria.

## Methods

### Diarrhoeagenic bacterial species

Test bacteria used in this study were diarrhoeagenic bacterial strains originally isolated from faecal and vomitous specimens of infants and children aged between 9 months and 1½ years of age, presenting for weaning diarrhoea at the Oni Memorial Children Hospital, Ibadan, Oyo State, Nigeria and at various residential homes [23]. Stock isolates were reactivated in sterile unbuffered peptone water (Lab M, Basingstoke, England) and incubated for 24-48 h at 35°C, after which they were sub-cultured by streaking on sterile plate count agar (PCA, Lab M, England) and MacConkey (MCC, Lab M, England) agar to assure purity. Pure isolates were stored on cystein lactose electrolyte deficient agar (CLED, Lab M, England) slants, while inoculum concentrations of the diarrhoeagenic bacteria used were 10^3^ cfu ml^-1^.

### Antibiotic susceptibility / resistance determination (paediatric antibiotic drugs)

Codes, names, active ingredients and manufactured / expiry dates of the 31 expired oral paediatric suspensions were as presented in Table 1, while those of the seven unexpired oral paediatric antibiotic suspensions were – 1. Throtal (erythromycin (125 mg /5ml): Maf. Date: Aug/2011: Exp. Date: Jul/2014); 3. Zinnat suspension (cefuroxime axetil (125 mg /5ml): Maf. Date: Jan/2011: Exp. Date: Jan/2013); 4. Tambac (cefpodoxime proxetil (50 mg/ 5ml): Maf. Date: Nov/2010: Exp. Date: Oct/2012); 5. Bactrim (sulfamethoxazole + trimethoprim (200 mg /5ml): Maf. Date: March/2011: Exp. Date: March/2016); 7. Loxaprim (co-trimoxazole (240 mg /ml): Maf. Date: Nov/2010: Exp. Date: Oct/2013); 8. Refucil (griseofulvin (125 mg /5ml): Maf. Date: June/2011: Exp. Date: May/2014); 10. Loxagyl suspension metronidazole (200 mg /5ml): Maf. Date: June 2011: Exp. Date: May 2013).

**Table 1:** Characteristic information of test oral paediatric antibiotics

Lab codes	Brand names of antibiotics	Active ingredients	Constitution (mg/5ml)	Manufactured Date	Expiry Date
1.	Throtal	Erythromycin	220	Oct. 2003	Sep. 2006
2.	Etocin	Erythromycin ethylsuccinate	200	Aug/2005	July/2008
3	Zinnat	Cefuroxime axetil	125	Mar/2007	Mar/2009
4	Tambac	Cefpodoxime proxetil	250	July/2003	July/2007
5	Bactrim	Sulfamethoxazole + Trimethoprim	200	May/2006	May/2011
6	Amoxycillin	Amoxycillin	228.5	Jan/2002	Jan/2005
7	Loxaprim	Co-trimoxazole	240	Jul/2006	Jun/2009
8	Refucil	Griseofulvin	125	Oct/2007	Oct/2010
9	Floxapen	Flucloxcacillin	125	June/2007	May/2010
10	Loxagyl	Metronidazole	200	June/2007	May/2009
11	Ampicillin	Ampicillin	125	June/2004	June/2007
12	Fleming	Amoxicillin + Clavulanic acid	125	Nov/2006	Nov/2008
13	Amoxycillin	Ampicillin trihydrate	125	Jan/2002	Jan/2005
14	Drimox	Amoxycilline	125	Sep/2004	Aug 2007
15	Amoksiklav	Amoxycillin + Clavulanic acid	228	Nov/2003	Nov/2005
16	Odoxil	Cefadroxil	250	Jan/2007	May/2010
17	Emzoclox	Ampicillin + Cloxacillin	250	Aug/2007	Jul/2010
18	Jawaclox	Ampicillin + Cloxacillin	250	Aug/2007	Jul/2010
19	Chloramphenicol	Chloramphenicol palmitate	125	Mar/2004	Feb/2007
20	Cefamor	Cefalexin	125	Sep/2006	Aug/2008
21	Zithromax	Azithromycin	200	Sep/2006	Sep/2008
22	Etocin	Erythromycin ethylsuccinate	200	May/2004	Apr/2007
23	Clofenicol	Chloramphenicol palmitate	125	Aug/2006	Aug/2009
24	Ceporex	Cephalexin	250	Jan/2004	Jan/2007
25	Erythrokid	Erythromycin	250	Nov/2006	Oct/2009
26	Vercef	Cefaclor	125	May/2006	Apr/2008
27	Sporidex	Cephalexin	250	Sep/2003	Aug/2006
28	Ampicillin	Ampicillin trihydrate	250	Jun/2004	Jun/2007
29	Ampicillin	Ampicillin trihydrate	125	July/2001	July/2004
30	Jawaclox	Ampicillin + Cloxacillin	250	Sept/2004	Aug/2007
31	Jawaclox	Ampicillin + Cloxacillin	250	Sept/2004	Aug/2007

Inhibitory activities of the 31 expired and seven unexpired oral paediatric antibiotic suspensions were determined on diarrhoeagenic bacterial strains metronidazole (200 mg/5ml): Maf. Date: June 2011: Exp. Date: May 2013).

Inhibitory activities of the 31 expired and seven unexpired oral paediatric antibiotic suspensions were determined on diarrhoeagenic bacterial strains, using a modification of agar well’ using a modification of agar well-diffusion method of Tagg et al [24]. Sterile Mueller-Hinton agar was poured into sterile Petri dishes and allowed to set. Wells of about 6.0 mm were bored into the agar followed by surface sterilisation of the agar plates by flaming. The entire surface of each of the cool sterile Mueller-Hinton agar plates was then seeded with each bacterial isolate and the plates were left for about 10 minutes before aseptically dispensing the paediatric antibiotic suspensions (antibiotic powder dissolved in recommended volume of sterile distilled water, added to sterile, plain semi solid agar), into the agar wells to avoid spreading of the antibiotic suspensions on the agar surfaces. The plates were incubated at 350C for 24-48 hours, and zones of inhibition were measured and recorded (triplicates) in millimetre diameter [25, 26], while zones less than 10.0 mm in diameter or absence of zones of inhibition were recorded as resistant (negative). For the current study, the adopted interpretation of susceptibility values were low = 10.0-19.0 mm; moderate = 20.0-29.0 mm; high = ≥ 30.0 mm.

## Results

Diarrhoeagenic bacterial strains of Staphylococcus aureus 11 (13.4%), E. coli 43 (52.4%), Klebsiella pneumoniae 16 (19.5%) and Proteus mirabilis 12 (14.6%) species assayed for in this study exhibited varying in vitro susceptibility and resistance patterns and profiles against 31 expired and seven unexpired oral paediatric antibiotic suspensions. Respective resistance rates against expired and unexpired oral paediatric antibiotic suspensions were Staphylococcus aureus (9.1-100% vs. 0.0-18.2%), E. coli (14.0 -100% vs. 9.1-15.9%), Klebsiella pneumoniae (0.0-100% vs. 0.0-31.3%) and Proteus mirabilis (8.4-100% vs. 25.0-41.7%) However, relatively high resistance rates of 45.5-55.8% (sulfamethoxazole + trimethoprim 5), 39.5-63.6% (amoxycillin 6), 46.5-54.5% (cotrimoxazole 7) and 37.5-63.6% (ampicillin + cloxacillin 18), as well as higher resistance rates of =75.0-100% towards the remaining expired antibiotics were generally exhibited.

Total percentage in vitro antibiotic resistance rates of between 25.6% (amoxycillin + clavulanate 15) and as high as =80.0%, with %MAR of 35.5-93.5% were exhibited by the E. coli strains against the expired antibiotics; although, lowest total percentage resistance of 14.0% and 16.3% respectively were exhibited against amoxillin + clavulanic acid 12 and ampicillin 11 (Table 1 and Table 2). Total percentage in vitro antibiotic resistance rates recorded among the E. coli strains against the seven unexpired oral paediatric antibiotics were between 9.1 and 15.9% but %MAR was 35.5-93.5% (Table 2 and Table 3). Total percentage in vitro resistance rates displayed by the Kleb. pneumoniae strains against the expired paediatric antibiotics were between 37.5 and 93.8%, while the lowest resistance rates were 6.3% (amoxillin + clavulanic acid 12) and 12.5% (amoxycillin + clavulanate 15) but all the Kleb. pneumoniae strains were totally resistant (100%) to erythromycin ethylsuccinate 22, ampicillin trihydrate 28, ampicillin 29, ampicillin + cloxacillin 30 and ampicillin + cloxacillin 31, although none was resistant to ampicillin 11, while %MAR was 51.6-83.9% (Table 1 and Table 3). Total percentage in vitro antibiotic resistance rates of the Kleb. pneumoniae strains against the seven unexpired oral paediatric antibiotics were 12.5-31.3% with%MAR of 28.6 – 57.2% (Table 2 and Table 3).

**Table 2:** *In vitro* antibiotic resistance patterns and susceptibility zones of inhibition profiles of diarrhoeagenic bacterial species against unexpired oral paediatric antibiotic suspensions

Bacterial species	1	3	4	5	7	8	10	% MAR
*E*. *coli*	15.9	15.9	11.4	11.4	9.1	13.7	15.9	28.6-100
	10.0-20.0	10.0-30.0	10.0-29.0	10.0-33.0	10.0-30.0	10.0-36.0	10.0-30.0	
*Klebsiella pneumoniae*	31.3	18.8	25.0	0.0	0.0	12.5	25.0	28.6-57.2
	10.0-19.0	10.0-23.0	10.0-20.0	10.0-30.0	10.0-30.0	10.0-30.0	10.0-28.0	
*Proteus mirabilis*	25.0	25.0	41.7	25.0	25.0	41.7	41.7	28.6-100
	10.0-25.0	10.0-17.0	10.0-19.0	15.0-30.0	10.0-29.0	10.0-28.0	10.0-20.0	
*Staphylococcus*	9.1	18.2	9.1	9.1	0.0	0.0	0.0	57.2
	10.0-20.0	10.0-20.0	10.0-29.0	10.0-30.0	10.0-28.0	10.0-28.0	10.0-28.0	
*Lactobacillus* spp.*	0.0	0.0	0.0	0.0	0.0	12.5	0.0	28.6
	11.0-20.0	10.0-18.0	11.0-27.0	10.0-30.0	10.0-33.0	10.0-30.0	10.0-29.0	

**Keys:** 1=Throtal (erythromycin); 3=Zinnat (cefuroxime axetil); 4=Tambac (cefpodoxime proxetil); 5=Bactrim (sulfamethoxazole + trimethoprim); 7=Loxaprim (cotrimoxazole); 8=Refucil (griseofulvin); 10=Loxagyl (metronidazole);% MAR =% multiple antibiotic resistance

* Non-diarrhoeagenic bacterial species

**% Total resistance of unexpired oral paediatric antibiotic suspensions (mg 5ml**^**-1**^**)**

**Table 3:** *In vitro* antibiotic resistance patterns and susceptibility zones of inhibition profiles of diarrhoeagenic bacterial species against expired oral paediatric antibiotic suspensions

Expired oral paediatric antibiotic suspensions (mg 5ml^-1^)																																
Bacterial species	1	2	3	4	5	6	7	8	9	10	11	12	13	14	15	16	17	18	19	20	21	22	23	24	25	26	27	28	29	30	31	%MAR
*E*. *coli*	**81.4**	**72.1**	**81.4**	**65.1**	**55.8**	**39.5**	**46.5**	**53.5**	**62.8**	**76.7**	**16.3**	**14.0**	**81.4**	**60.5**	**25.6**	**95.3**	**95.3**	**53.5**	**65.1**	**74.4**	**74.4**	**83.4**	**93.0**	**90.7**	**86.1**	**72.1**	**81.4**	**100**	**93.0**	**93.0**	**97.8**	**35.5-93.5**
	10.0-15.0	10.0-20.0	10.0-20.0	10.0-18.0	10.0-30.0	10.0-22.0	10.0-27.0	10.0-24.0	10.0-25.0	10.0-21.0	11.0-30.0	10.0-29.0	10.0-25.0	10.0-20.0	10.0-25.0	10.0-20.0	10.0-22.0	10.0-28.0	10.0-25.0	10.0-26.0	10.0-21.0	10.0-22.0	10.0-15.0	10.0-25.0	10.0-20.0	10.0-20.0	10.0-23.0	-	10.0-18.0	10.0-17.0	10.0	
*Kleb*. *pneumoniae*	**87.5**	**68.8**	**87.5**	**68.8**	**50.0**	**56.3**	**50.0**	**75.0**	**75.0**	**87.5**	**0.0**	**6.3**	**81.3**	**56.3**	**12.5**	**93.8**	**87.5**	**37.5**	**68.8**	**68.8**	**87.5**	**100**	**81.3**	**81.3**	**75.0**	**62.5**	**93.8**	**100**	**100**	**100**	**100**	**48.4-83.9**
	10.0-12.0	11.0-20.0	10.0-19.0	10.0-16.0	10.0-21.0	10.0-20.0	10.0-20.0	23.0-25.0	10.0-20.0	10.0-11.0	10.0-30.0	10.0-30.0	10.0-15.0	10.0-22.0	10.0-23.0	11.0	10.0-11.0	16.0-25.0	14.0-24.0	10.0-25.0	10.0-17.0	-	10.0-11.0	10.0-16.0	10.0-20.0	10.0-28.0	10.0	-	-	-	-	
*Prot*. *mirabilis*	**58.4**	**33.3**	**83.3**	**66.6**	**50.0**	**51.7**	**50.0**	**75.0**	**51.7**	**75.0**	**16.7**	**8.4**	**66.7**	**66.7**	**16.7**	**75.0**	**75.0**	**50.0**	**66.7**	**83.3**	**75.0**	**75.0**	**91.7**	**66.7**	**75.0**	**58.3**	**75.0**	**91.7**	**91.7**	**91.7**	**100**	**19.4-93.5**
	10.0-20.0	10.0-25.0	11.0	10.0-12.0	13.0-20.0	11.0-20.0	10.0-20.0	11.0-22.0	13.0-21.0	10.0-16.0	17.0-28.0	15.0-35.0	10.0-20.0	10.0-20.0	11.0-21.0	10.0-20.0	10.0-22.0	10.0-20.0	10.0-25.0	12.0-20.0	10.0-21.0	10.0-20.0	20.0	10.0-20.0	10.0-21.0	13.0-21.0	11.0-20.0	21.0	20.0	20.0	-	
*Staph*. *aureus*	**81.8**	**54.5**	**81.8**	**72.7**	**45.5**	**63.6**	**54.5**	**45.5**	**54.5**	**81.8**	**18.2**	**9.1**	**100**	**90.9**	**45.5**	**90.9**	**100**	**63.6**	**90.9**	**72.7**	**81.8**	**100**	**90.9**	**90.9**	**81.8**	**81.8**	**90.9**	**100**	**100**	**100**	**100**	**45.2-93.5**
	10.0-18.0	10.0-18.0	10.0-20.0	10.0-17.0	10.0-25.0	10.0-18.0	15.0-18.0	10.0-16.0	10.0-16.0	10.0-12.0	10.0-30.0	10.0-26.0	-	15.0	13.0-25.0	10.0	-	13.0-16.0	10.0	12.0-25.0	10.0	-	10.0	15.0	10.0-18.0	13.0-24.0	10.0	-	-	-	-	
*Lactobacillus* spp.*	**75.0**	**75.0**	**100**	**62.5**	**37.5**	**62.5**	**50.0**	**62.5**	**75.0**	**75.0**	**37.5**	**25.0**	**75.0**	**87.5**	**50.0**	**87.5**	**100**	**62.5**	**50.0**	**50.0**	**75.0**	**62.5**	**75.0**	**50.0**	**37.5**	**50.0**	**62.5**	**100**	**87.5**	**87.5**	**100**	**45.2-93.5**
	10.0	12.0-25.0	-	10.0-23.0	10.0-25.0	10.0-26.0	10.0-30.0	12.0-27.0	11.0-24.0	16.0-25.0	13.0-26.0	20.0-30.0	10.0-17.0	20.0	12.0-25.0	19.0	-	10.0-26.0	10.0-23.0	10.0-26.0	12.0-14.0	10.0-15.0	10.0-13.0	10.0-17.0	10.0-22.0	14.0-28.0	10.0-14.0	-	10.0	10.0	-	

**Keys:** 1 = erythromycin; 2 = erythromycin ethylsuccinate; 3 = cefuroxime axetil; 4 = cefpodoxime proxetil; 5 = sulfamethoxazole + trimethoprim; 6 = amoxycillin; clavulanic acid; 13 = amoxycillin trihydrate; 14 = amoxycilline; 15 = amoxycillin + clavulanate; 16 = cefadroxil; 17 = ampicillin + cloxacillin; 18 = ampicillin + cloxacillin; chloramphenicol; 24 = cephalexin; 25 = erythromycin; 26 = cefaclor; 27 = cephalexin; 28 = ampicillin trihydrate; 29 = ampicillin; 30 = ampicillin + cloxacillin

* Non-diarrhoeagenic bacterial species

Total percentage in vitro resistance rates of Proteus mirabilis strains towards expired antibiotics was between 33.3% (erythromycin ethylsuccinate 2) and 91.7% (chloramphenicol 23, ampicillin trihydrate 28; ampicillin 29; ampicillin + cloxacillin 30) but all the strains were resistant to ampicillin + cloxacillin 31. Lowest resistance rates of 8.4%, 8.4% and 16.7% were however, exhibited against amoxillin + clavulanic acid 12, ampicillin 11 and amoxycillin + clavulanate 15 respectively, while %MAR was 45.2-93.5% (Table 1 and Table 3). Total percentage in vitro antibiotic resistance rates of the Pr. mirabilis strains against the seven unexpired oral paediatric antibiotic suspensions were 25.0-41.7% with %MAR of 28.6-100% (Table 2 and Table 3).

All the Staph. aureus strains were totally resistant to expired amoxycillin trihydrate 13, ampicillin + cloxacillin 17, erythromycin ethylsuccinate 22, ampicillin trihydrate 28; ampicillin 29, ampicillin + cloxacillin 30, ampicillin + cloxacillin 31, while lowest resistance were 9.1% (amoxillin + clavulanic acid 12) and 18.2% (ampicillin 11) but higher resistance rates of between 45.5% (sulfamethoxazole + trimethoprim 5), (griseofulvin 8), (amoxycillin + clavulanate 15) and 90.9% (amoxycilline 14, cefadroxil 16, chloramphenicol 23, cephalexin 24, 27) were exhibited against the remaining expired paediatric antibiotics. The %MAR of 54.8–93.5% was recorded among the Staph. aureus strains (Tables 1 and 3). Total percentage in vitro antibiotic resistance patterns of the Staph. aureus strains against the seven unexpired oral paediatric antibiotic suspensions were between 9.1 and 18.2%, while %MAR was 57.2% (Table 2 and Table 3).

Eight strains of Lactobacillus species, which were non-diarrhoeagenic but served as control also exhibited total percentage resistance of 50.0 – 87.5% towards the expired paediatric antibiotics, while t all the strains were resistant to expired cefuroxime axetil, 3, ampicillin + cloxacillin 17, ampicillin 28 and ampicillin + cloxacillin 31. Total percentage resistance of 25.0% (amoxillin + clavulanic acid 12) and 37.5% (ampicillin 11) were the least recorded, with MAR of 45.2–77.4% (Tables 1 & 3). None of the Lactobacillus strains was resistant to unexpired erythromycin 1, cefuroxime axetil 3, cefpodoxime proxetil 4, sulfamethoxazole + trimethoprim 5, cotrimoxazole 7 and metronidazole 10 but 12.5% total resistance was recorded against unexpired griseofulvin 8. None of the Lactobacillus strains exhibited total susceptibility towards the expired antibiotics but all the strains exhibited multiple antibiotic susceptibility towards the unexpired antibiotics, except a strain of Lactobacillus sp. which exhibited %MAR of 28.6%, i.e., 12.5% antibiotic resistance each towards Refucil (griseofulvin 8) and Loxagyl (metronidazole 10) (Table 4).

**Table 4:** Overall and comparative total *in vitro* percentage multiple antibiotic resistance rates and profiles of the diarrhoeagenic bacterial species towards the expired and unexpired paediatric antibiotics

% Total resistance of expired / unexpired antibiotics (mg 5ml^-1^)											
Bacterial species	Type	1	25	3	4	5	7	8	10	% MAR	% Multiple antibiotic resistance profiles
*E. coli* [43]	E	81.3	86.1	81.4	65.1	55.8	46.5	53.5	76.7	35.5-93.5	35.5 [1], 38.7 [2], 51.6 [1], 54.8 [1], 58.1 [4], 61.3 [1], 64.5 [4], 67.7 [7], 71.0 [4], 74.2 [4], 77.4 [3], 80.6 [6], 83. 9 [2], 87. 1 [1], 90.3 [1], 93.5 [1]
	U	15.9		15.9	11.4	11.4	9.1	13.7	15.9	28.6-100	28.6 [4], 57.2 [2], 71.2 [2], 100 [2]
*K. pneumoniae* [16]	E	87.5	75.0	87.5	68.8	50.0	50.0	75.0	87.5	48.4-83.9	48.4 [1], 51.6 [1], 61.3 [1], 64.5 [1], 67.7 [1], 71.0 [1], 74.2 [1], 77.4 [1], 80.6 [2], 83.9 [3]
	U	31.3		18.8	25.0	0.0	0.0	12.5	25.0	28.6-57.2	28.6 [2], 42.9 [2], 57.2 [2]
*Pr*. *mirabilis* [12]	E	58.4	75.0	83.3	66.6	50.0	50.0	75.0	75.0	19.4-93.5	19.4 [1], 45.2 [2], 48.4 [1], 64.5 [1], 71.0 [1], 74.2 [1], 80.6 [3], 93.5 [1]
	U	25.0		25.0	41.7	25.0	25.0	41.7	41.7	28.6-100	28.6 [3], 57.2 [2], 71.4 [1], 100 [1]
*S. aureus* [11]	E	81.8	81.8	81.8	72.7	45.5	54.5	45.5	81.8	45.2-93.5	45.2 [1], 58.1 [1], 61.3 [1], 64.5 [1], 67.7 [2], 74.2 [1], 83.9 [2], 90.3 [1], 93.5 [1]
	U	9.1		18.2	9.1	9.1	0.0	0.0	0.0	57.2	57.2 [1]
*Lactobacillus* spp. [8]*	E	75.0	37.5	100	62.5	37.5	50.0	62.5	75.0	45.2-93.5	45.2 [1], 48.4 [1], 51.6 [1], 61.3 [1], 77.4 [1], 80.6 [2], 93.5 [1]
	U	0.0		0.0	0.0	0.0	0.0	12.5	12.5	28.6	28.6 [1]

**Keys:** 1 Throtal (erythromycin); 3 Zinnat (cefuroxime axetil); 4 Tambac (cefpodoxime proxetil); 5 Bactrim (sulfamethoxazole + trimethoprim); 7 Loxaprim (cotrimoxazole); 8 Refucil (griseofulvin); 10 Loxagyl (metronidazole), 25=(erythromycin); E=expired; U=unexpired.=non-diarrhoeagenic bacterial species

Widest zones of inhibition (in mm diameter) among the diarrhoeagenic bacterial species towards expired and unexpired oral paediatric antibiotic suspensions were closely similar, E. coli (25.0-33.0 vs. 29.0-33.0), Kleb. pneumoniae (25.0-33.0 vs. 25.0-35.0), Staph. aureus (25.0-30.0 vs. 25.0-30.0), Lactobacillus strains (25.0-33.0 vs. 25.0-33.0) but more of the bacterial strains exhibited widest zones of inhibition towards the unexpired antibiotics than expired antibiotics (Table 5).

**Table 5:** Overall comparative zones of inhibition patterns and profiles of the diarrhoeagenic bacterial species towards the expired and unexpired paediatric antibiotics

Bacterial species	Type	% Zones of inhibition profiles
*E. coli*	E	10.0 [115], 11.0 [19], 12.0 [29], 13.0 [37], 14.0 [18], 15.0 [39], 16.0 [13], 17.0 [7], 18.0 [20], 19.0 [23], 20.0 [33], 21.0 [9], 22.0 [8], 23.0 [11], 24 [5], 25.0 [12 (2.9%)], 26.0 [2 (0.5%)], 27.0 [3 (0.7%)], 28.0 [1 (0.2%)], 29.0 [2 (0.5%)], 30.0 [2 (0.5%)], 33.0 [1 (0.2%)] ≥ 25.0 [23 (5.6%)] **S [409 (30.7%)] / R [924 (69.3%)]**
	U	10.0 [80], 11.0 [19], 12.0 [9], 13.0 [7], 14.0 [7], 15.0 [9], 16.0 [5], 17.0 [11], 18.0 [7], 19.0 [15], 20.0 [21], 21.0 [6], 22.0 [7], 23.0 [10], 24 [1], 25.0 [13 (5.0%)], 26.0 [2 (0.8%)], 27.0 [8 (3.1%)], 28.0 [5 (1.9%)], 29.0 [7 (2.7%)], 30.0 [3 (1.2%)], 33.0 [1 (0.4%)] ≥ 25.0 [39 (15.0%)]* **S [260 (86.4%)] /R [41 (13.6%)]**
*Kleb. Pneumoniae*	E	10.0 [35], 11.0 [7], 12.0 [3], 13.0 [7], 14.0 [2], 15.0 [17], 16.0 [7], 17.0 [7], 18.0 [7], 19.0 [6], 20.0 [21], 21.0 [2], 22.0 [4], 23.0 [3], 24.0 [3], 25.0 [7 (4.9%)], 26.0 [1 (0.7%)], 28.0 [1 (0.7%)], 29.0 [2 (1.4%)], 30.0 [1 (0.7%)], 33.0 [1 (0.7%)] ≥ 25.0 [13 (9.0%)] **S [144 (29.0%)] /R [352 (71.0%)]**
	U	10.0 [31], 11.0 [6], 13.0 [5], 14.0 [3], 15.0 [8], 16.0 [2], 19.0 [8], 20.0 [4], 22.0 [5], 23.0 [2], 24 [1], 25.0 [4 (4.3%)], 26.0 [1 (1.1%)], 27.0 [3 (3.2%)], 28.0 [4 (4.3%)], 29.0 [2 (2.2%)], 30.0 [4 (4.3%)] ≥ 25.0 [18 (18.4%)]* **S [93 (83.0%)] /R [19 (17.0%)]**
*Pr*. *mirabilis*	E	10.0 [28], 11.0 [8], 12.0 [6], 13.0 [11], 14.0 [5], 15.0 [8], 16.0 [5], 17.0 [2], 18.0 [2], 19.0 [4], 20.0 [33], 21.0 [6], 22.0 [2], 23.0 [2], 24 [1], 25.0 [4 (3.0%)], 26.0 [1 (0.75%)], 28.0 [2 (1.5%)], 29.0 [1 (0.75%)], 30.0 [1 (0.75%)], 35.0 [1 (0.75%)] ≥ 25.0 [10 (7.5%)] **S [133 (35.8%)] /R [239 (64.2%)]**
	U	10.0 [12], 11.0 [3], 12.0 [1], 13.0 [4], 14.0 [2], 15.0 [4], 15.0 [4], 16.0 [1], 17.0 [3], 18.0 [2], 19.0 [4], 20.0 [5], 21.0 [2], 22.0 [1], 23.0 [1], 25.0 [2 (3.4%)], 27.0 [5 (8.6%)], 28.0 [1 (1.7%)], 29.0 [3 (5.2%)], 30.0 [1 (1.7%)] ≥ 25.0 [12 (20.7%)]* **S [58 (69.0%)] /R [26 (31.0%)]**
*Staph. aureus*	E	10.0 [26], 12.0 [5], 13.0 [10], 14.0 [5], 15.0 [9], 16.0 [10], 17.0 [1], 18.0 [7], 19.0 [1], 20.0 [5], 21.0 [2], 23.0 [2], 24.0 [2], 25.0 [4 (4.3%)], 26.0 [1 (1.1%)], 27.0 [1 (1.1%)], 29.0 [2 (2.2%)], 30.0 [1 (1.1%)] ≥ 25.0 [9 (9.7%)] **S [93 (27.3%)] /R [248 (72.7%)]**
	U	10.0 [21], 11.0 [2], 12.0 [1], 13.0 [3], 14.0 [1], 15.0 [4], 16.0 [4], 17.0 [4], 18.0 [3], 19.0 [6], 20.0 [5], 22.0 [1], 23.0 [1], 24 [3], 25.0 [2 (2.8%)], 26.0 [1 (1.4%)], 27.0 [3 (4.2%)], 29.0 [1 (1.4%)], 30.0 [1 (1.4%)] ≥ 25.0 [8 (11.1%)]* **S [72 (93.5%)] /R [5 (6.5%)]**
*Lactobacillus* spp.	E	10.0 [18], 11.0 [5], 12.0 [4], 13.0 [5], 14.0 [4], 15.0 [6], 16.0 [5], 17.0 [2], 18.0 [2], 19.0 [2], 20.0 [6], 21.0 [2], 22.0 [2], 23.0 [3], 24 [1], 25.0 [5 (6.2%)], 26.0 [5 (6.2%)], 27.0 [1 (1.2%)], 28.0 [1 (1.2%)], 30.0 [3 (3.7%)] ≥ 25.0 [15 (18.5%)]* **S [81 (32.7%)] / R [167 (67.3%)]**
	U	10.0 [10], 11.0 [5], 12.0 [2], 13.0 [1], 14.0 [1], 15.0 [7], 16.0 [4], 17.0 [5], 18.0 [1], 19.0 [2], 20.0 [1], 24 [1], 25.0 [3 (5.6%)], 27.0 [1 (1.9%)], 29.0 [1 (1.9%)], 30.0 [3 (5.6%)], 33.0 [1 (1.9%)] ≥ 25.0 [9 (16.7%)] **S [54 (96.4%)] / R [2 (3.6%)]**

**Keys:** 1=Throtal (erythromycin); 3=Zinnat (cefuroxime axetil); 4=Tambac (cefpodoxime proxetil); 5=Bactrim (sulfamethoxazole + trimethoprim); 7 Loxaprim (co-trimoxazole); 8 Refucil (griseofulvin); 10 Loxagyl (metronidazole), 25=(erythromycin); E=expired; U=unexpired.

* Higher rates of widest zones of inhibitions

None of the 31 expired paediatric antibiotics or the seven unexpired antibiotics assayed for in the current study was protected with desiccants at the time of purchase.

## Discussion

Presented global estimates of child deaths due to diarrhoea abound [23, 27–30] but major therapeutic intervention for diarrhoea consists of fluid and electrolyte therapy [31], especially as part of treatment strategies adopted in the treatment of infantile diarrhoea, which is usually by administering oral rehydration salts (ORS) solution [32–35]. In some cases of infantile diarrhoea (gastroenteritis) however, anti-diarrhoeal drugs are administered [23]; while in most cases, antibiotics are administered on infants and children, even before they are taken to the hospital. When antimicrobial therapy is appropriate for diarrhoeal cases, selection of a specific antimicrobial agent is expected to be made based upon susceptibility patterns of the pathogen or information on local susceptibility patterns [31]. But in spite of this suggestion, high prevalence of antibiotic resistance is still commonly reported as a continually increasing problem that has, to a greater or lesser extent, affected virtually every area of the world, especially in clinical paediatric conditions [23, 36–38].

Since a law was passed in 1979, drug manufacturers are required to stamp an expiration date on their products, which is the date that the manufacturer can still guarantee full potency and safety of the drug, so, it turns out that the expiration date on a drug does stand for something, contrary to what some think [39]. Comparing the rates of antibiotic resistance and multiple antibiotic resistance in this study, it was observed that higher total percentage antibiotic resistance and multiple antibiotic resistance rates were mostly recorded against expired antibiotics than the unexpired antibiotics. Apart from Proteus mirabilis strains, which exhibited 41.7% resistance against the unexpired antibiotics, only 0.0-25.0% resistance rates were generally recorded among other diarrhoeagenic bacteria, compared to 0.0-100% resistance rates exhibited towards the expired antibiotics. Even among the non-diarrhoeagenic Lactobacillus strains, which served as control, similar higher resistance and multiple antibiotic resistance rates were recorded for the expired antibiotics. The grievous implication is that usage of such expired antibiotics knowingly or unknowingly, not only can lead to antibiotic resistance in vivo but more likely can also lead to degradation of their active ingredients into components that can be seriously hazardous; thereby, causing child deaths due to treatment failure during clinical therapeutic intervention for infantile diarrhoea.

More wider zones of inhibition were generally recorded for the unexpired antibiotics. But considering the zones of inhibition profiles between the expired and unexpired oral paediatric antibiotics used in this study, and the fact that two of the 31 expired antibiotic suspensions were comparatively bacteriostatic in vitro, even after their expiration dates, one may want to ponder on the argument that antibiotics are still potent after the expiration dates. However, there is a strong possibility that potency of the two expired antibiotics based on in vitro susceptibility may vary considerably in vivo. Thus, a patient may feel better for some time after taking such supposedly potent expired antibiotics but will ultimately come down with the infection or with even worse clinical condition. In addition, many antibiotics, being heat- and moisture-labile are particularly vulnerable to degraded active constituents, especially when stored for a very long period of time at inappropriate temperatures and humidity [4–6, 40, 41], such as when drug consignments are exposed to such adverse conditions during shipment [42] or at tropical ports, while they await lengthy port clearance.

Another implication of significant resistance rates recorded for expired paediatric antibiotics in this study is that when most drugs reach their expiration dates, they will start to taste/smell/look different, and since antibiotics are also particularly time-sensitive drugs, the primary concern is that the active drug compounds will break down over time and turn into different chemicals that can be more difficult for the human body to metabolise. Liquid medications like paediatric antibiotics are usually mixed with preservatives; thus, when the expiration date is breached, the preservatives can no longer work properly, and the chemical compositions of the drugs begin to break down, sometimes forming dangerous by-products. Specifically, these by-products can harm the kidney and liver, and when those organs are impaired, the body loses the ability to filter toxins and various chemical metabolites from the system, which can constitute more health hazard, especially in the immune system [14]. These by-products can even be carcinogenic, which if extrapolated as additional clinical failure, can lead to mortality; therefore, strict compliance with expiry dates ought not to be ignored.

None of the paediatric antibiotics assayed for in the current study was protected with desiccants, including the unexpired antibiotics. Medications without protective coatings are usually packaged with desiccants or little packets of silica gel, which prevents any moisture in the air from acting on them, so as to prevent them from losing their potency. Keeping these medications after their expiration dates may cause them to start taking in moisture, which in turn can induce microbial contamination [43]. Drugs that do not comply with minimum standards are illegal in all countries but the quality of many antibiotics and other drugs in some developing countries, including Nigeria is often below standards in the formulary [14, 22].

It is a known practice that expiry dates are sometimes changed, and this hazardous practice is common among some drug peddlers and unscrupulous individuals or groups of backyard drug manufacturers. Quite a number of press coverage had reported various incidences of massive infant deaths in Nigeria due to consumption of bad quality drugs, unfortunately, since analytical laboratories to detect substandard drugs are generally uncommon in developing countries, therapeutic failure is usually the only indication of expired or substandard drugs. There were no available data to further discuss the microbial, clinical and public health significance of administration and consumption of expired antibiotics in Nigeria but findings of this study can serve as preliminary basis and confirmation data to elucidate the fact that expired antibiotics are not of any clinical importance, and therefore, should be banned from being sold and administered. Just as in the cases of adulterated antibiotics, expired antibiotics can also lead to therapeutic failure due to poor quality, such as drug degradation, microbial contaminations and medication toxicity [44–46]; thereby, producing sub-inhibitory concentrations in vivo, which can ultimately cause increase in the selection of antibiotic resistant bacterial strains, as well as induced drug allergies.

## Conclusion

Misuse or abuse of antibiotics in clinical practice alone cannot explain the high frequency of resistant bacteria in developing countries [47–49]. Thus, a missing link of an additional reason for antibiotic resistance is provided by the findings of this study, in which significantly low bacteriostatic potentials, as well as high antibiotic resistance were recorded among expired oral paediatric antibiotics. Administrations of expired antibiotics are a kind of over-looked but certain significant cause of high antibiotic resistance in clinical cases and possibly in other cases; therefore, attention should be drawn to impending hazards of expired antibiotics. It is also necessary for developing countries like Nigeria to have effectively implemented judicious national policies that will ban sales, as well as ensure non-administration and non-consumption, in any form, of expired antibiotics and other expired medications, most especially in paediatric cases. Effective enforcement of the World Health Organisation (WHO) guidelines on drug donations can also curtail such practices [50]. All these can serve as means of justifying advocacy against sales and administration of expired antibiotics, most especially in paediatric cases.
